# Enhancing Stress-Resistance for Efficient Microbial Biotransformations by Synthetic Biology

**DOI:** 10.3389/fbioe.2014.00044

**Published:** 2014-10-20

**Authors:** Haiyang Jia, Yanshuang Fan, Xudong Feng, Chun Li

**Affiliations:** ^1^Department of Biological Engineering, School of Life Science, Beijing Institute of Technology, Beijing, China

**Keywords:** synthetic biology, stress-resistance, stress condition, biotransformation, robustness

## Abstract

Chemical conversions mediated by microorganisms, otherwise known as microbial biotransformations, are playing an increasingly important role within the biotechnology industry. Unfortunately, the growth and production of microorganisms are often hampered by a number of stressful conditions emanating from environment fluctuations and/or metabolic imbalances such as high temperature, high salt condition, strongly acidic solution, and presence of toxic metabolites. Therefore, exploring methods to improve the stress tolerance of host organisms could significantly improve the biotransformation process. With the help of synthetic biology, it is now becoming feasible to implement strategies to improve the stress-resistance of the existing hosts. This review summarizes synthetic biology efforts to enhance the efficiency of biotransformations by improving the robustness of microbes. Particular attention will be given to strategies at the cellular and the microbial community levels.

## Introduction

In the biotechnology industry, chemicals required for a wide variety of sectors including food, medicine, and energy can be obtained through microbe-mediated chemical conversions, alternatively known as “microbial biotransformations.” Despite the versatile applications, the full potential of microbial biotransformations has not been fulfilled yet due to several unsolved problems (Woodley, 2013). One critical problem is that the growth and productivity of host organisms are often severely constrained by multiple adverse conditions, which lead to stress. Therefore, exploring methods to improve the stress tolerance of organisms within the fermentation industry, could significantly improve the productivity, shrink production costs, reduce energy consumption, increase substrate utilization, mitigate the risk of contamination, and so on.

Synthetic biology, defined as the application of engineering principles to biology, aims to make the process of designing genetically encoded biological systems more systematic, predictable, robust, scalable, and efficient (Wang et al., 2013). Consequently, by supplying cells with devices and parts that confer resistance to stress, synthetic biology therefore offers a powerful approach for improving the properties of existing microbial biotransformation systems. In this review, we will describe recent synthetic biology efforts to improve the robustness of microbes for the purpose of increasing biotransformational efficiencies. Particular focus will be given to improvements made on the cellular and the microbial community levels.

## Environment Stress and Cellular Stress Response Mechanisms

Cellular stress response is a general term covering a wide range of molecular changes that cells undergo in response to environmental stressors, including extreme temperature, high salt condition, strongly acidic solution, toxins, and mechanical damage. These stresses can often inhibit cell growth and division, reduce cell viability, cause abnormal morphology of cells, destroy plasma membrane structure/function (Davidson et al., 1996), disturb metabolism (Eleutherio et al., 1995), and repress the synthesis of many proteins (Walker and Van Dijck, 2006). A classic example of these is the fermentation of bioethanol, an alternative fuel that has drawn much attention due to energy and environmental concerns. A major challenge in bioethanol fermentation is that the yields and titers from the microbial fermentation are usually held back by the accumulation of ethanol toxicity (Fischer et al., 2008; Wackett, 2008). In order to maintain optimal conditions for metabolite production and for the survival of the microbial host, it is often necessary to have in place feedback control systems. However, such measures can significantly increase the overall cost of the biotransformational process.

In nature, microorganisms have a variety of mechanisms for evolutionary adaptations and physiological acclimation that allow them to survive and remain active in the face of environmental stress. The various processes involved in cellular stress responses serve the adaptive purpose of protecting a cell against unfavorable environmental conditions, both through short term mechanisms that minimize acute damage to the cell’s overall integrity, and through long-term mechanisms, which provide the cell with a measure of resiliency against similar adverse conditions (Welch, 1993). Functionally, stress-inducible proteins can be grouped into seven classes, which function in the following areas: (1) helping proteins folding or refolding as molecular chaperones (Ellis, 1990), (2) clearing misfolded and irreversibly aggregated proteins (Tyedmers et al., 2010), (3) curing non-physiological covalent modifications of nucleic acids (Jantschitsch and Trautinger, 2003), (4) reorganizing and stabilizing the energy and metabolism supply of the cells (Macario et al., 2006), (5) initiating the stress response pathways or inhibiting expression cascades (Al Refaii and Alix, 2009), (6) sustaining cellular structures such as the cytoskeleton (An et al., 2004), and (7) transporting, detoxifying, and membrane-modulating (Welker et al., 2010).

## Enhancing Resistance at Cellular Level

Through thousands of years of evolution, microbes have acquired a number of self-protective mechanisms in order to survive and adapt to a range of environments. In particular, microbes living in extreme environments, known as extremophiles, can provide us with great insights for developing robust hosts.

### Heat-shock proteins for minimizing enzyme destabilization

Molecular chaperones, including the heat-shock proteins (Hsps), are a ubiquitous feature of cells, which cope with stress-induced denaturation of cellular proteins, Studies of Hsps in model organisms undergoing experimental stress have shown that: (a) expression of Hsps can occur in nature (Lindquist, 1986), (b) all species have Hsp genes, which vary in their expression patterns, (c) Hsps expression can be correlated with resistance to stress (Richter et al., 2010), and (d) species thresholds for Hsps expression are correlated with levels of stress that they naturally undergo (Feder and Hofmann, 1999). In order to reduce the toxicity of product and by-product accumulation arising from biotransformations (Papoutsakis, 2008; Nicolaou et al., 2010; Dunlop, 2011), GroESL, as one type of Hsps, was over expressed with its natural promoter, and the tolerance of the host to alcohols was greatly enhanced, including a 12-fold increase in total growth in 48 h culture under 4% (v/v) ethanol, a 2.8-fold increase under 0.75% (v/v) *n*-butanol, a 3-fold increase under 1.25% (v/v) 2-butanol, and a 4-fold increase under 20% (v/v) 1,2,4-butanetriol (Zingaro and Terry Papoutsakis, 2013). Owing to the adaptability of stress–response genes during long-term natural evolution, extremophilic bacteria exhibit superior robustness under harsh conditions (Stetter, 1999; Egorova and Antranikian, 2005; Podar and Reysenbach, 2006). Identification and introduction of such genes from extremophiles has proved to be an effective approach for engineering the cellular robustness of microbes (Pan et al., 2009; Dunlop et al., 2011; Lin et al., 2013). The thermotolerance and ethanol tolerance of *E. coli* were significantly enhanced by overexpressing the GroESL from the solvent-tolerant *Pseudomonas putida*. Also, GroESL from the thermophilic *Thermoanaerobacter tengcongensis* endowed *Clostridium acetobutylicum* with improved corn cob hydrolyzates (CCH)-tolerance as well as elevated butanol productivity (Luan et al., 2014). In another study, heat-shock genes from thermophiles were designed, introduced, and screened in *Saccharomyces cerevisiae* to improve its thermotolerance. Among 10 engineered thermotolerant yeasts, T.te-TTE2469, T.te-GroS2, and T.te-IbpA displayed over 25% increase of cell density and 1.5- to 4-fold of cell viability compared with the control, and metabolic rates were also improved in the engineered thermotolerant strain. The engineered *Saccharomyces cerevisiae* for β-amyrin fermentation showed 28% increased β-amyrin titer with a broadened growth temperature range and a reduced fermentation period (Liu et al., 2014). In addition, the heterologous expression of the 17 kDa small Hsp from the nematode *Caenorhabditis elegans*, CeHSP17, enabled *E. coli* to grow at 50°C, which is the highest growth temperature ever reported (Ezemaduka et al., 2014).

### Efflux pumps for alleviating metabolite toxicity

Microbes have several strategies for addressing biofuel toxicity (Isken and De Bont, 1998; Ramos et al., 2002). Efflux pumps, a class of transmembrane protein complexes that export toxins from the cell based on a mechanism of active transport, play an important role in the resistance of cells toward solvent (Putman et al., 2000; Nikaido and Takatsuka, 2009). In Gram-negative bacteria, transporter complexes involved in metabolite efflux such as AcrAB-TolC are typically composed of three protein components: (i) an inner membrane protein responsible for substrate recognition, (ii) a periplasmic linker, and (iii) an outer membrane channel. Several solvent-resistant efflux pumps have been characterized, such as ttgABC, ttgDEF, ttgGHI (Rojas et al., 2001) and srpABC (Kieboom et al., 1998), which displayed varying tolerance to different solvents. Recently, a systematic approach was established to screen a library of primarily uncharacterized heterologous pumps for engineering biofuel-tolerant host strains. In total, 43 pumps were heterologously expressed in *Escherichia coli*, and their resistance against seven representative biofuels was tested. With a competitive growth assay, positive pumps with improved survival capacity were efficiently distinguished (Dunlop et al., 2011). To increase the efficiency of efflux, directed evolution was applied to reconstruct native transporters, which allowed a rapid selection of AcrB variants showing enhanced efflux of linear and cyclic fuel molecule candidates, *n*-octane and α-pinene, and two positive mutants were isolated exhibiting increased efflux efficiency for *n*-octane and α-pinene by up to 47 and 400%, respectively (Foo and Leong, 2013). Besides, nonane (C9), decane (C10), and undecane (C11) as significant toxicants accumulated in *Saccharomyces cerevisiae* can induce a range of cellular behaviors such as efflux pumps, membrane modification, radical detoxification, and energy supply. The possible role of efflux pumps in alkane secretion further strengthens their use for the development of alkane-tolerant microbes (Ling et al., 2013). By exploiting ABC transporters in *Yarrowia lipolytica*, the heterologous expression of ABC2 and ABC3 transporters were used to increase the tolerance against decane and undecane in *Saccharomyces cerevisiae* through maintaining lower intracellular alkane level (Chen et al., 2013). Furthermore, to improve ethanol tolerance of *Saccharomyces cerevisiae*, a presumed multidrug/solvent efflux pump, ADP1 from *Saccharomyces cerevisiae* BY4741, was cloned and overexpressed in the same strain. Overexpression of the gene allowed the engineered strain to show higher specific growth rate and greater ethanol productivity compared to its parental strain in the presence of 5–7.5% (v/v) ethanol. The ethanol productivity and amount were 20–50% higher than those of the wild-type strain (Yang et al., 2013).

### Transcriptional regulators for exerting global changes

Regulation of gene transcription is fundamentally important for microbes to maintain normal cellular activity and respond to environmental changes. Through the intricate but efficient transcriptional regulatory networks, microbes can quickly respond to transitory environmental changes (e.g., pH, temperature, nutrition, and osmotic pressure), and optimize their metabolism to adapt to new conditions (Gasch et al., 2000; Browning and Busby, 2004). In recent years, several research groups have attempted to directly or indirectly manipulate the transcriptional regulatory network by engineering transcriptional regulatory proteins to enhance microbial stress tolerance in industrial application. With an increasing number of regulators being identified and characterized, this approach is promising for the purpose of stress tolerance improvement (Lin et al., 2013). Haa1 is a transcriptional activator required for the adaptation of *Saccharomyces cerevisiae* to weak acids. Through the constitutive overexpression of HAA1, the host strain acquired a higher level of acetic acid tolerance (Tanaka et al., 2012). EvgA and YdeO (two transcriptional regulators) were subsequently linked together to enhance heat resistance (Christ and Chin, 2008). The survival test indicated that the *evgA* amplification can improve thermal tolerance by 10^2^–10^4^ fold. This study suggested an overlap in the transcriptional regulatory network could control the thermal and fatty acids resistance. Indeed, the genes induced by EvgA under acidic conditions were found to be similar to genes induced by high temperature after long-term exposure (Masuda and Church, 2003; Riehle et al., 2003). IrrE from an extreme radiation-resistant bacterium, *Deinococcus radiodurans*, was also recently engineered to improve resistance against multiple stresses in *E. coli*. Constitutive expression of the *irrE* gene in *E. coli* promoted DNA repair and protected the host against oxidative, osmotic, and thermal damage (Pan et al., 2009). Though the wild-type IrrE protein cannot confer higher ethanol or butanol tolerance to *E. coli*, a laboratory-evolved IrrE mutant was selected, which enhanced host tolerance to multiple chemicals, including ethanol, butanol, isobutanol, pentanol, isopentanol and acetate (Chen et al., 2011), 1-butanol (Zhang et al., 2012), and isobutanol (Chong et al., 2014). Further to this, ethanol (Chong et al., 2013) tolerance of *E. coli* was also greatly enhanced through random mutagenesis of global transcription factor cyclic AMP receptor protein (CRP).

## Enhancing Resistance at Biological Population Level

In nature, apart from individual cell adaption, interaction within the whole cell population also plays a critical role during adaption to stressful conditions. This cooperative behavior seemingly supports the altruistic adage of “together we are everything, alone we are nothing.” Thus, engineering microbial community behavior is potentially a promising strategy for developing robust, stress-resistant microbes.

### Engineering cell to cell communication to program altruistic death

Programed death is generally associated with bacterial response to stressful conditions. *Salmonella typhimurim* responds to competition in the host’s microbiota by causing host inflammation through programed death in order to kill the microbiota and reduce competition (Stecher et al., 2007; Ackermann et al., 2008). *E. coli* responds to amino-acid starvation by triggering programed death in order to provide nutrients to surviving cells (Aizenman et al., 1996). Colicinogenic *E. coli* responds to DNA-damaging agent and nutrient depletion by releasing colicin through cell lysis, so as to kill neighboring competitors (Gardner et al., 2004; Cascales et al., 2007). These behaviors appear paradoxical as they offer no benefit to the individual. A common explanation is that the death is “altruistic” whereby the death of cells can help to keep the bacterial community in a viable state (West et al., 2007).

Recently, by engineering tunable, stress-induced altruistic death in *E. coli*, the conditions under which bacterial programed death became advantageous were investigated experimentally. The engineered *E. coli* carried two extra genetic circuits. The first one was a programed cell death module where a lytic enzyme was induced by the beta-lactam antibiotic 6-aminopenicillanic acid (6-APA). The antibiotic levels were modulated manually to provide a tunable environmental stress. The second module was an IPTG-inducible beta-lactamase (BlaM) to improve resistance toward the environmental stress by degrading 6-APA. Importantly, BlaM was produced and stored intracellularly, and only released upon lysis, thus making cell death beneficial for the remaining survivors. Assisted by a mathematical model, the researchers predicted the optimal programed death rate that maximized population growth under stress (Tanouchi et al., 2012).

### Engineering microbial consortia to perform complicated tasks

Microbial consortia can perform even more complicated tasks and endure more dynamic environments than monocultures (Brune and Bayer, 2012). They may compliment or inhibit mutually in a mixed culture that flourish under various environmental constraints and conditions (Lee et al., 2013; Smid and Lacroix, 2013). Most natural microbial ecosystems are usually extremely complex in terms of their ecological structures, interaction patterns, and fluctuating environmental and evolutionary stresses, thus making it quite challenging to rationally engineering and optimize these complicated ecosystems. Furthermore, consortia can be more robust to environmental fluctuations due to two organizing features. First, members of the consortium communicate with each other by trading metabolites or by exchanging dedicated molecular signals. The second feature refers to the division of labor, and the overall output of the consortium depends on a combination of tasks performed by constituent individuals or subpopulations (Brenner et al., 2008).

Two strains of *E. coli* were engineered to metabolize glucose and xylose such that they consumed these substrates at similar rates. When grown in co-culture, the two strains fermented the sugars more efficiently than either single engineered strain (Eiteman et al., 2008). In addition, they were able to resist nutrient limitation stress better because of the diversity of metabolic modes available to the mixed species combined with the ability to share metabolites within the community (Fay, 1992). Living within a community is conducive for generating robustness toward environmental fluctuations and enhancing the stability of the members in a consortium. Compared with monocultures, communities might be more capable of resisting the invasion of other species (Burmølle et al., 2006). Seventeen epiphytic bacterial strains were screened for synergistic interactions within biofilms in different combinations. Four isolated strains were found to function synergistically in biofilms. When exposed to the antibacterial agent hydrogen peroxide or tetracycline, the relative activity of the four-species biofilms was markedly higher than that of any single-species biofilms. Moreover, the four-species biofilms performed better in resisting invasion than the single-species biofilms (Briones and Raskin, 2003). Also functional strains *Zoogloea resiniphila* and two other uncultured strains, *Acinetobacter* sp. clone JT2 and bacterium clone P1D1-516 can tolerate the organic loading rate (Adav et al., 2009). Engineered consortia may perform most reliably in a changeable environment when various metabolic modes are present among members (Kato et al., 2008). The synthetic consortium consisting of *K. vulgare* and *B. Megaterium* has been successfully applied in a two-step fermentation process for the industrial production of vitamin C (Song et al., 2014). With an integrated time-series, proteomic and metabolomic analysis of the industrial production process of vitamin C, a synergetic phenomenon was observed in which the sporulation of *B. megaterium* was found to promote the growth of *K. vulgare* and its production of 2-keto-gulonic acid, via the supply of essential purines. Also, the released proteins upon cell lysis of *B. megaterium* conferred *K. vulgare* resistance to the stress from reactive oxygen species (ROS). It also revealed that the transmembrane transport of substrates (in particular, thiamine and glutathione) into the cell was achieved by the oligopeptide transport system (thiBPQ), which thus enhanced the tricarboxylic acid cycle and pentose phosphate pathway. This process is likely to generate more ATP and NADPH, which could then be utilized in combating against intracellular ROS (Ma et al., 2011, 2012).

## Concluding Remarks

Biotransformations permit the production of chemicals in an economic and environment-friendly way. Despite its versatility, such processes have still not yet fulfilled their potential. This is due in part to the vulnerability of host organisms to fluctuating environmental conditions. Synthetic biology offers scientists tremendous opportunities to engineer microbial strains with desired properties to tolerate these conditions. Implementation of stress-resistant traits leading toward increased robustness is an attractive strategy for improving the efficiencies of biotransformations. More efforts are still required to increase understanding of the stress-resistant mechanisms exhibited in nature. In addition, future attention needs to be given to the screening and engineering of natural plug-and-play stress-resistant modules and development of more feasible methods for the re-design of the host chassis. Overall, the development of highly robust microbial hosts systems will require an in-depth understanding of stress-resistant mechanisms operating at both the cellular and community levels.

## Conflict of Interest Statement

The authors declare that the research was conducted in the absence of any commercial or financial relationships that could be construed as a potential conflict of interest.
